# Perceptions and Knowledge About the MenB Vaccine Among Parents of High School Students

**DOI:** 10.1007/s10900-020-00954-1

**Published:** 2021-01-02

**Authors:** Eric Richardson, Kathleen A. Ryan, Robert M. Lawrence, Christopher A. Harle, Alyson Young, Melvin D. Livingston, Amit Rawal, Stephanie A. S. Staras

**Affiliations:** 1 Department of Health Outcomes and Biomedical Informatics, College of Medicine, University of Florida, Gainesville, FL USA; 2 The Institute for Child Health Policy, University of Florida, 2004 Mowry Road, Room 2238, Gainesville, FL 32610 USA; 3 Department of Pediatrics, College of Medicine, University of Florida, Gainesville, FL USA; 4 Rollins School of Public Health, Emory University, Atlanta, GA USA; 5 Avnee Foundation, Gainesville, FL USA

**Keywords:** Immunization, MenB, Community intervention, Cross-sectional survey, Focus group

## Abstract

Serogroup B meningococcal disease (MenB) causes almost 60% of meningitis cases among adolescents and young adults. Yet, MenB vaccine coverage among adolescents remains below 10%. Since parents are the primary medical decision makers for adolescents, we examined MenB vaccination rates and parent attitudes about meningitis and the MenB vaccine. In 2018, in conjunction with a county-wide, school-based immunization campaign, we conducted a mixed methods study among parents of 16- to 17-year-olds. We facilitated focus groups asking parents about their knowledge of meningitis and reactions to educational materials and sent behavioral surveys based on Health Belief Model constructs to parents through the county high school system. Parents in three focus groups (n = 8; participation rate = 13%) expressed confusion about their child’s need to receive the MenB vaccine in addition to the meningococcal conjugate vaccine (MenACWY), but conveyed strong trust in their physicians’ recommendation. Among survey participants (n = 170), 70 (41%) had heard of the MenB vaccine. Among those 70 parents, the most common barriers to vaccination were concerns about side effects (55%) and uncertainty of susceptibility due to receipt of the MenACWY vaccine (30%). The percentage of teens that received at least one dose of the MenB vaccine was 50% (n = 35) by parent report and 23% (n = 16) by state vaccination records. Parents demonstrated uncertainty and confusion about the MenB vaccine particularly due to the existence of another meningitis vaccine and limited health care provider recommendations. Confirmatory studies of parent confusion about the MenB vaccine are needed to develop interventions.

Between 2014 and 2016, serogroup B (MenB) was the cause of 58% of the 166 cases of meningococcal disease in persons aged 18 to 24 years in the United States [1, 2]. Notably, serogroup B is the primary cause of organization-based outbreaks and has an approximate case fatality rate of 7% [3, 4]. The best strategy to prevent MenB disease is vaccination with one of the two vaccines licensed for use among 10- to 25-year-olds [5]. As of 2016, the Advisory Committee on Immunization Practices’ (ACIP) recommends either of the MenB vaccines as a shared clinical decision (Category B) for healthy individuals between 16- and 23-years (preferred at 16- to 18-years) and routine (Category A) to individuals ages ≥ 10-years-old who are at increased risk (e.g., persistent complement component deficiencies, complement inhibitor use, asplenia or during disease outbreaks) [6]. In 2019, MenB vaccine initiation (i.e., received at least one dose) among 16- to 18-year-olds in the United States was an estimated 21% [7].

Since parents are often the primary decision makers for adolescent vaccines, parental acceptance of the MenB vaccine is likely key to increasing uptake [8]. A handful of studies performed outside the United States have assessed parents’ perceptions about the MenB vaccine and found most parents have heard of meningitis (80–86%) and wanted their child to receive the MenB vaccine (62–64%) [9–12]. Within the United States, to our knowledge, only two studies examined parents’ awareness about and intention for their high-school-aged children to receive the MenB vaccine [13, 14]. Among a 2017 convenience sample of 445 parents of Minnesota high school students, 72% had heard of meningitis, but only 20% were aware of the MenB vaccine [13]. In this study, compared to parents who had not heard of the vaccine, the odds of parents’ intent to have their child vaccinated increased nearly fourfold among parents aware of the MenB vaccine and threefold among parents at least somewhat concerned about meningitis. A second study included a random sample of 619 parents across the US, and weighted results to correspond with the US population [14]. Based on the weighted results, an estimated 43% of US parents are aware of the MenB vaccine, and among those only 45% intend to get the MenB vaccine. As such, only an estimated 19% of parents intended to receive the MenB vaccine.

Theory and vaccination studies suggest, however, that parental awareness of a vaccine is not sufficient to lead to vaccination [15–19]. A widely recognized behavioral theory, the Health Belief Model suggests parents' decision to get their child vaccinated is influenced by: (1) perceived threat and severity of the disease, (2) belief the vaccine will offer benefits (e.g., preventing meningitis), (3) perceived barriers to getting the vaccine, (4) self-efficacy to complete the behavior, and (5) a cue to action (e.g., a recommendation from a healthcare provider). Guided by the Health Belief Model, we aimed to identify rates and parent attitudes about MenB vaccination for 16- to 17-year old adolescents. We chose 16- to 17-year-olds because they are within the recommended age of vaccination and are dependent upon parent consent to receive the vaccine. Understanding more about parent attitudes could help develop and adapt interventions to increase MenB vaccination and ultimately reduce MenB meningitis cases.

## Methods

### Research Design

We used a mixed method design with parent focus groups and a cross-sectional survey to understand parents’ attitudes about the MenB vaccine. The study included parents of 16- to 17-year-olds in a north-central Florida county, Alachua. The University of Florida Institutional Review Board approved study procedures.

### Focus Groups

In February 2018, we invited parents of 16- to 17-year-old adolescents residing in Alachua County who had visited a University of Florida primary care clinic in the past year to participate in focus groups. We called parents up to three times at phone numbers obtained through a registry of University of Florida patients who consented to receiving research invitations [20]. We reminded agreeing parents on the day before the focus groups.

Immediately before the focus groups, we orally explained the informed consent to the group of parents. Parents who agreed to participate signed the informed consent form and participated in the focus groups. A trained moderator conducted the groups in a private room by following a semi-structured guide. Questions included parents’ opinions about meningococcal disease, the MenB vaccine, and the content and design of publicly available education materials from the Centers for Disease Control and Prevention, Immunization Action Coalition, and National Meningitis Association [21–23]. Incentives included a meal and $25. Focus groups were audio-recorded and transcribed.

### Qualitative Analysis

We conducted a thematic analysis of focus group transcripts using Nvivo qualitative data analysis software Version 12 [24]. Between December 2019 and March 2020, two members of the research team (AY, ER) analyzed the focus group transcripts using thematic analysis from three rounds of independent coding [25]. In the first coding round, we created two primary themes. In the second coding round, we assigned each response to a primary theme and identified secondary themes. After conferring about the saliency of the secondary themes, we created a coding framework. In the third coding round, we applied the coding framework to the transcripts. We selected specific quotes to represent each coded theme.

### Cross-Sectional Survey

In March, May and November 2018, we invited parents to complete a survey through the county school system. Surveys were collected before and after a county-wide, school-based vaccination campaign. In April and May 2018, the Florida Department of Health sent vaccination consent forms to parents of students in 11th and 12th grades (approximate ages 16 to 18 years) via the county school superintendent’s office distribution list. With a signed parent consent, students received immunizations for MenB, meningococcal conjugate vaccine (MenACWY), human papillomavirus (HPV), and hepatitis A on specified days at county high schools.

In March 2018, prior to the vaccination campaign, we randomly selected one-third of the county’s 10–12th grade English classes to distribute 1250 survey packets including a waiver of documentation of consent letter, paper survey, $1 cash, and a stamped return envelope. English teachers distributed packets to students in assigned classes. The waiver of documentation of consent letter explained the purpose of the study and all elements of informed consent. The letter instructed parents that returning the completed survey would be considered as providing consent. Returned paper-based surveys were double entered into Research Electronic Data Capture (REDCap) [26, 27].

In May 2018, due to low participation rates in March 2018, we sent a second invite to parents. Parents of high school students were invited via the county school superintendent’s office distribution list to complete the survey using either their packet or a link to an online, REDCap survey. Parents who completed the survey online signed an electronic consent form in REDCap.

In November 2018, while originally planned as a time for a follow-up survey of spring participants, due to low participation rates in March and May, we again invited all high school parents to complete the survey. The superintendent’s office used their distribution list to invite all parents to complete the online, REDCap survey.

### Survey Constructs

Using items adapted from previous vaccine surveys (e.g., the Health Information National Trends Survey and the National Survey of Children’s Health), we assessed the theoretical constructs of the Health Belief Model [19, 28–30]. We measured perceived severity by participants’ familiarity with meningitis and if it posed a serious threat to their child. We asked participants if meningitis caused four outcomes (loss of limbs, death, brain damage, and cancer). We created a composite knowledge score representing the total number of correctly identified outcomes ranging from 0 (all incorrect) to 4 (all correct).

Among parents who had heard of the MenB vaccine, we measured the perceived benefits and barriers of the MenB vaccine. A potential barrier considered was prior receipt of the MenACWY vaccine because of its potential confusion with MenB and its Category A recommendation for routine vaccination at 11 years and a second dose at 16 years from the ACIP [31]. Lastly, we assessed provider recommendations using three questions from prior surveys [19, 32]: whether a healthcare provider discussed, recommended, and, if recommended, expressed importance.

### MenB Vaccine Initiation

We assessed vaccine initiation with two imperfect data sources: parent-report and vaccination records in the Florida immunization registry [33]. Adapted from the National Survey of Children’s Health [28], we asked parents to report whether their adolescent received MenACWY vaccine, meningococcal B (MenB) vaccine, both, or not sure. To objectively measure vaccine initiation, we requested parents provide identifying information for the child. Using this information, the Florida Department of Health staff obtained records of MenB vaccine doses received from the state immunization registry by the approximate date parents completed the survey.

### Quantitative Analysis

We conducted a descriptive analysis of the survey data. To maintain independence of responses we used the first response for parents who completed the survey more than once (n = 5). We compared the sensitivity and specificity of parent-reported vaccines to the Florida immunization registry records using a crosstab analysis. Analyses were conducted using SAS software version 9.4 (SAS Institute, Inc., Cary, NC).

Because of the small number of respondents prior to the Department of Health campaign (n = 40), we did not attempt to evaluate changes in parent attitudes or vaccination rates based on the campaign. We were, however, concerned that parents who completed the survey after the vaccination campaign may have been influenced by the campaign. To assess for this potential influence, we compared knowledge of the MenB vaccine in parents who responded prior to the campaign (March 2018) to those who responded after the campaign (May and November 2018).

## Results

### Focus Groups

Among the 61 parents with contact information in the research registry, 8 attended one of three focus group sessions (the groups included 3, 3, and 2 participants, respectively) for a 13% participation rate. Participating parents were mainly educated with at least a college degree (5/8), non-Hispanic white (5/8), and between 36- and 52-years-old (5/8). All of the parents reported that their child had an established primary care pediatrician. Focus groups lasted an average of 70 min (Range 63–84 min).

Most parents were aware of meningitis or meningococcal disease. Common descriptions about the disease included ‘serious,’ ‘fast,’ and ‘devastating.’ For example, one parent described meningitis as: “I mean when you see – hear the stories about kids in college, amputations, all these consequences and that, right, then you realize, well, it may be a small chance that it’s going to happen, but when it happens it’s just devastating.”

When reviewing MenB vaccine educational materials, parents expressed confusion about meningococcal vaccines. Parents asked why children needed to get a second vaccine for meningitis and why serogroup B had a separate vaccine. Exemplar quotations were:

Because they provide routine vaccinations at age 11 to 12 for all the rest, right? So it's like maybe, well, my child's good enough, they've got that vaccine, so why do I have to give him another vaccine for another type of meningitis?

It's just one serial group that it covers. And it says other meningococcal vaccines are recommended to help protect against the ACWY. So I'm just wondering why is this one – is this one just more common?

By and large, parents reported they would follow the advice of their pediatrician regarding vaccines. For example, one parent said: “ I mean, not being in the medical field, I feel like that's something I'm going to trust my pediatrician to choose and talk to me about the right medicine to use.”

### Survey Respondents

Overall, 170 caregivers of 16–17-year-olds completed our survey at three time points: 40 in March (prior to the Department of Health campaign), 46 in May, and 84 in November 2018 Most respondents (97%) were parents; thus, for the remainder of the paper, we refer to caregivers as parents. Parents were an average of 49 years-old (range 34–69 years), and majority were non-Hispanic white (78%), educated (28% receiving a Bachelor’s degree and 33% receiving a Master’s degree or above), married (75%), and had insurance for their child (96%) (Table 1). In our sample, approximately half (56%) had a female 16- to 17-year-old.

**Table 1:** Demographic characteristics of parents who completed the survey

	Participants (N = 170)
	N (%)
Race/ethnicity	
White non-Hispanic	128 (78%)
Hispanic or Latino/a	13 (8%)
African American or Black (non-Hispanic)	10 (7%)
Other	9 (6%)
Education	
High school diploma or less	20 (5%)
Vocational degree	5 (9%)
Associates degree	34 (17%)
Bachelor’s degree	39 (28%)
Master’s degree or higher	57 (33%)
Child’s health insurance	
Through current or former employer	89 (71%)
Medicaid, medical assistance	12 (10%)
Other	8 (5%)
Purchased directly from insurance company	7 (6%)
Not covered by any insurance	5 (4%)
Marital status	
Married	117 (75%)
Divorced	16 (11%)
Never married	8 (5%)
Widowed	5 (3%)
Not married, living with partner	5 (3%)
Separated	4 (3%)

### Awareness of Meningitis and the Meningitis Vaccines

Among the 170 parents, almost all (94%) had heard of meningococcal disease or meningitis. When asked about meningococcal vaccines, parents were more likely to report having heard about MenACWY (61%) than the MenB vaccine (40%) (χ^2^ = 79.19 p < 0.001). We found no evidence of temporal increases in awareness potentially due to the Department of Health campaign that would preclude combining parent responses: similar percentages of parents had heard of the MenB vaccine in March 2018 (35%) as in May and November 2018 (42%) (χ^2^ = 2.13 p = 0.71).

### Perceived Seriousness of Meningitis

Regardless of having heard about the MenB vaccine, almost all (96%) parents agreed that meningitis posed a serious threat to their child, and correctly identified death and brain damage as meningitis outcomes (Table 2). About half of parents correctly identified that cancer was not an outcome of meningitis. Correct identification of loss of limbs as an outcome of meningitis was more common among parents that heard of MenB vaccine (41%) than parents who had not heard of the MenB vaccine (21%) (p = 0.01). When comparing overall composite knowledge scores, the parents who had heard of the MenB vaccine had a 0.3 higher average score than parents that had not heard of the MenB vaccine (p = 0.03).

**Table 2:** Differences in knowledge about meningitis based on awareness of MenB vaccine

Do you think meningococcus can cause…	Heard of MenB vaccine (N = 66)	Not heard of MenB vaccine (N = 80)	p-value
	Answered correctly	Answered correctly	
Death	65 (98%)	74 (93%)	0.88
Brain or nerve problems	63 (95%)	77 (96%)	0.85
Cancer	40 (61%)	43 (54%)	0.40
Loss of limbs	27 (41%)	17 (21%)	0.01
Mean Composite Score	3.0	2.7	0.03

### Perceived Benefits, Barriers, and Susceptibility

**Fig. 1:**
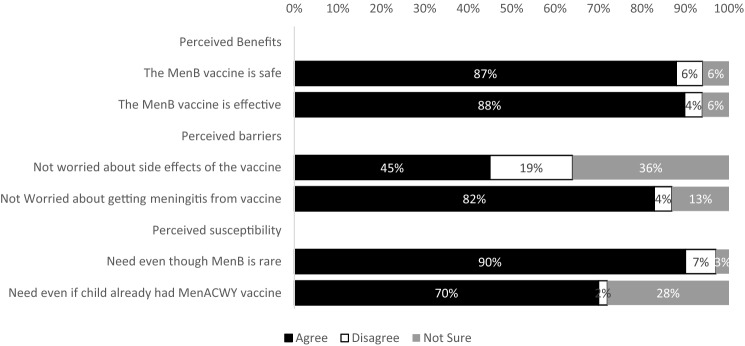
Parents’ attitudes about the MenB vaccine (n = 70)

Among parents who had heard of the MenB vaccine (n = 70), most parents agreed that the benefits of the MenB vaccine included safety and effectiveness (Fig. 1). Perceived barriers centered on side effects: 19% were concerned and 36% were unsure. Since their child had received the MenACWY vaccine, about one-quarter of parents (28%) were unsure, and 2% thought their child did not need the MenB vaccine.

### Provider Recommendation

Most parents (86%) reported their child attended at least one preventative visit in the previous year. Among parents who heard of the MenB vaccine (n = 70), 81% indicated that a healthcare provider spoke to them about the MenB vaccine, and 75% reported that their doctor recommended it. A majority (71%) indicated that receiving the vaccine was important to their provider. Assuming that all parents who have not heard of MenB did not receive a recommendation from their healthcare provider (0/100), only 31% (52/170) of participating parents may have received a recommendation for the MenB vaccine by a healthcare provider.

### MenB Vaccine Initiation

All parents that had heard of the MenB vaccine (n = 70) provided their child’s information to allow us to obtain records from the state vaccine registry. The Department of Health staff found vaccination records for all 70 children. Based on the state immunization registry records, 16/70 (23%) of the 16- to 17-year-olds had received at least one dose of the MenB vaccine. But, based on parent-report, 35/70 (50%) had received at least one dose of the MenB vaccine. To estimate the MenB vaccination rates for all respondents, we assumed that none of the children of parents who were unaware of MenB vaccine had received the vaccine. Under this assumption, between 9% (16/170) and 22% (37/170) of the 16- to 17-year-olds had received at least one dose of the MenB vaccine. Among the 16 adolescents with records of at least one dose of the MenB vaccine, 56% completed the series.

When we compared parent-reported vaccination to the immunization registry, the child’s MenB vaccine status agreed just over half of the time (Table 3). Among the 16 children who had records of receiving the MenB vaccine, only ten parents reported the child had received the vaccine (sensitivity = 0.63). Among the 54 children without records for the MenB vaccine, 25 parents reported their child had received the vaccine (specificity = 0.46).

**Table 3:** Sensitivity and specificity of parents’-report MenB vaccine initiation (N = 70)

	State immunization registry			
	Vaccine	No vaccine	Total	
Parents’-report				
Vaccine	10	25	35	PPV = 0.29
No vaccine	6	29	35	NPV = 0.17
Total	16	54	70	
	Sensitivity = 0.63	Specificity = 0.46		Correct classification = 56%

## Discussion

In addition to typical barriers for vaccination, parents expressed uncertainty about the relationship between the two meningitis vaccines; a sentiment that potentially impedes MenB vaccine uptake. Similar to other new vaccines, only half of the parents had heard of the MenB vaccine and the majority of those who heard about it were concerned about side effects [34]. Unlike other vaccines, parents expressed concerns about susceptibility to meningitis and the need for the MenB vaccine since their child already received a meningitis vaccine, MenACWY. Finally, while parents expressed confusion about the need for an additional meningitis vaccine, they ultimately expressed trust in their physician.

As with other adolescent vaccines when newly recommended, a majority of parents were not aware of the MenB vaccine. For example, in 2007, only 42% of parents could identify the MenACWY vaccine as a recommended vaccine [34]. By 2017, the awareness of the MenACWY vaccine increased to 75% [13]. Some researchers attributed low parental awareness of MenACWY to infrequent physician recommendations and missed clinical opportunities [35]. A similar phenomena may be occurring for MenB vaccine. Nearly all parents in our study reported that their child had at least one preventive visit during the past year, and therefore, had an opportunity to receive the MenB vaccine. Under the assumption that none of the parents who were unaware of the MenB vaccine had received a provider recommendation or their child had received the vaccine, an estimated 31% of parents received a physician recommendation and 9–22% had initiated the MenB vaccine. These results suggest that 70–80% of 16- to 17-year-olds had missed opportunities to receive the MenB vaccine.

The large proportion of parents concerned about MenB vaccine side effects is also similar to other adolescent vaccines suggesting that between 41 and 73% of parents are concerned about vaccine side effects [19, 36]. Literature on vaccine hesitancy shows that concern about side effects is a significant barrier to parents deciding whether their adolescent receives a vaccine [37–39]. As such, the concern about MenB vaccine side effects may reflect a general concern about vaccines.

Parents who had heard of the MenB vaccine were unsure about their child’s susceptibility to MenB: an important predictor of vaccination according to the Health Belief Model [18]. Over two times as many parents were concerned about their child’s susceptibility to MenB compared to MenACWY (< 10% of a nationally representative sample of parents) [40, 41]. The higher percentage concerned and reports from our focus groups suggest that the time sequencing of the meningococcal vaccines (MenACWY recommended at 11- to 12-year-olds and MenB recommended for 16- to 17-year-olds) may inadvertently cause parents to perceive MenB vaccine as the other or less important meningitis vaccine.

Parents’ confusion about the relationship between the two vaccines is also reflected in the low sensitivity and specificity we found between parent-reported and immunization registry recorded MenB vaccination. The 56% match found between parent report and immunization records is well below other studies showing 78% to 88% match rates between parent report and immunization records for adolescent vaccines [40–42]. Parents may think receiving the meningococcal conjugate vaccine will protect their child from meningitis in general or simply be unaware that there are two distinct meningococcal vaccines.

The observed parents’ confusion about the MenB vaccine may also be a reflection of physician uncertainty. A national survey found that over half (59%) of pediatricians found it difficult to explain category B recommendations to parents, and as many as 12% provided information about the conjugate vaccine when recommending the MenB vaccine [43, 44]. The resulting unclear communication limits the inherent power of physician vaccine recommendations [45–47].

Our study includes three important limitations. First, our survey sample size was small due low participation rates. The sample size was reduced further for many questions since only half of parents had heard of the MenB vaccine. Moreover, our sample may not be representative of parents of Alachua county high school students or other areas of the United States. Second, because we had low response rates to our initial survey collection, we did not analyze separate time points including pre-/post- differences of the Florida Department of Health vaccination campaign. Without evidence of a temporal increase in awareness of the MenB vaccine during our study period, we analyzed the data as a cross-sectional evaluation of parents’ attitudes and adolescents’ vaccination rates. Third, our focus groups included a limited number of parents and we primarily targeted understanding the acceptability of nationally available education materials. However, these materials did include information relevant to perceptions about the MenB vaccine.

This study has three important strengths. First, our study is one of a few for MenB vaccine that specifically targets parents of high-school-aged children [13]. This understudied group of late adolescents represents an important opportunity for teens to receive the MenB vaccine before college entry and increased exposure. Second, the study includes an objective measure of MenB vaccine receipt. Previous research on parents’ attitudes about the MenB vaccine relied on self-report measures about parental intent to vaccinate their child [13]. Third, we included both quantitative and qualitative data to examine parents’ perceptions and knowledge. The qualitative data provided nuanced information about parents’ confusion about the vaccine and trust in the provider, while the survey data provided a sense of the relative frequency of specific hesitations.

Among parents of 16- to 17-year-olds, uncertainty about the relationship between the two meningitis vaccines likely presents a barrier to MenB vaccine uptake. While several of the parent concerns were similar to those expressed for other vaccine, unlike other adolescent vaccines, parents were unsure about the susceptibility of their child to MenB primarily because of the MenACWY vaccine. Future studies should consider how physicians can discuss the MenACWY and MenB vaccines with parents in ways that present a clear message of the importance of both vaccines.
